# Correlation of chemokines and growth factors with radiation-induced liver injury after interstitial high dose rate (HDR) brachytherapy of liver metastases

**DOI:** 10.1007/s00432-022-04041-x

**Published:** 2022-05-21

**Authors:** Robert Damm, Maciej Pech, Paola Cavalli, Florian Haag, Severin Gylstorff, Jazan Omari, Maximilian Thormann, Ricarda Seidensticker, Jens Ricke, Max Seidensticker, Borna Relja

**Affiliations:** 1 Department of Radiology and Nuclear Medicine, Otto-von-Guericke-University, Magdeburg, Germany; 2 Radiology Practice, Dessau, Germany; 3 Research Campus STIMULATE, Otto-von-Guericke University, Magdeburg, Germany; 4 Experimental Radiology, Department of Radiology and Nuclear Medicine, Otto-von-Guericke-University, Leipziger Strasse 44, 30120 Magdeburg, Germany; 5 Department of Radiology, Ludwig-Maximilians-University, Munich, Germany

**Keywords:** MCP-1, CX3CL1, VEGF, NGF, Liquid biopsy, Biomarker

## Abstract

**Background:**

Locoregional therapies, as imaging-guided tumor-directed procedures, are emerging treatment strategies in the management of primary and secondary liver malignancies such as e.g. colorectal cancer liver metastases. As one of those, irradiation-based interstitial high dose rate brachytherapy (iBT) of liver metastases bears a risk of developing focal radiation-induced liver injury (fRILI). Since little is known about biological factors involved in hepatic dysfunction after irradiation, the aim of this study was to identify factors, that may play a role in the underlying mechanism of fRILI, and that potentially may serve as biomarkers for post-therapeutic fRILI to improve specific management and treatment of patients.

**Methods:**

Twenty-two patients with hepatic malignancies (tumor patients, TP) underwent iBT with total ablative doses of radiation to the target volume ranging from e.g. 15 to 25 Gy. Hepatobiliary magnetic resonance imaging (MRI) was performed 6 weeks after iBT to quanitify fRILI. Blood samples were taken before (pre) and 6 weeks after (post) iBT from TP, and from ten healthy volunteers (HV controls) for the analyses of humoral mediators: monocyte chemoattractant protein-1 (MCP-1), chemokine (C-X3-C motif) ligand 1 (CX3CL1), vascular endothelial growth factor (VEGF) and beta-nerve growth factor (beta-NGF) using the Multi-Analyte Flow Assay via flow cytometry. Correlation analyses between the humoral mediators (pre and post iBT) with the tumor volume and fRILI were performed.

**Results:**

While MCP-1 and CX3CL1 tended to decrease in TP vs. HV, VEGF was significantly decreased in TP vs. HV pre and post iBT (*p* < 0.05). Beta-NGF levels were significantly increased in TP vs. HV pre and post iBT (*p* < 0.05). Baseline circulating levels of MCP-1, VEGF and beta-NGF have shown significant positive correlations with the hepatic tumor volume (*p* < 0.05). Circulating levels of humoral mediators before treatment did not correlate with fRILI, while CX3CL1 and VEGF after iBT have shown significant positive correlations with fRILI (*p* < 0.05).

**Conclusion:**

Tumor volume and threshold dose of irradiation damage correlated positively with MCP-1 and VEGF as well as NGF and CX3CL, respectively. Thus, investigation of biological mediators in blood samples from tumor patients may provide an appropriate tool to predict fRILI after interstitial HDR brachytherapy of liver metastases.

## Introduction

Local-ablative therapies are emerging treatment options for primary and secondary liver malignancies such as hepatocellular carcinoma or colorectal cancer liver metastases (Vogel et al. 2021; Cutsem et al. 2016). In the last two decades, several techniques have been developed including thermal and irradiation-based ablation therapy, such as e.g. Yttrium^90^ radioembolization (Y90 RE), stereotactic-ablative body radiotherapy (SABR) and high dose rate (HDR) interstitial brachytherapy (iBT) (Garza-Ramos and Toskich 2021; Ricke and Wust 2011; Manchec et al. 2021; Romesser et al. 2021). A common limitation of the liver-directed radiotherapy is radiation-induced liver disease (RILD), often originating from sinusoidal obstruction syndrome (SOS), formerly known as hepatic veno-occlusive disease (VOD) (Fan and Crawford 2014; Bayraktar et al. 2017). Histopathological findings of RILD include deposits of extracellular matrix leading to a congestion of sinusoids and central veins with subsequent necrosis of hepatocytes (Lawrence et al. 1995; Fajardo and Colby 1980). In case that liver dysfunction induced by irradiation exceeds a tolerable limit, clinical symptoms of RILD such as weight gain and jaundice by hyperbilirubinemia occur (Sangro et al. 2008; Doi et al. 2017). RILD is stratified in two types: classic RILD and non-classic RILD. Patients with classic RILD usually exert fatigue, increased abdominal girth, abdominal pain, hepatomegaly and anicteric ascites approximately 2 weeks to 4 months after hepatic irradiation (Lawrence et al. 1995). The pathological hallmark is VOD. While the levels of alkaline phosphatase increase, the levels of transaminases and bilirubin remain normal (Liang et al. 2011). Non-classic RILD develops in patients who suffer from chronic hepatic diseases, e.g. cirrhosis and viral hepatitis (Cheng et al. 2004). Patients with non-classic RILD exert more dysregulated hepatic functions with jaundice and/or, in contrast the classic RILD, markedly increased levels of transaminases rather than alkaline phosphatase (Cheng et al. 2004; Pan et al. 2010; Kim and Jung 2017). Compared with loco-regional treatment by Y90 RE, SABR or iBT provide high-conformal irradiation of liver malignancies and offer the possibility to quantify focal radiation-induced liver injury (fRILI), which is visualized in hepatobiliary magnetic resonance imaging (MRI) by fusion with the 3D irradiation plan (Hass et al. 2019). This technique facilitates the correct assessment of the threshold isodose of hepatic irradiation tolerance. Regarding molecular mechanisms of hepatic dysfunction after irradiation only little is known. Although the knowledge derives from analyses of VOD/SOS after bone-marrow transplantation (BMT), this is not necessarily representative for conditions induced by SABR and iBT (Shulman and Hinterberger 1992).

Although our knowledge of fRILI pathogenesis has improved in the last years, the underlying molecular pathomechanisms remain unclear. The irradiation causes early DNA damage, oxidative stress and the production of reactive oxygen species, which lead to hepatocellular apoptosis and acute inflammatory responses in irradiated regions (Yahyapour et al. 2018; Baran et al. 2022; Robbins and Zhao 2004). During the past decade considerable developments of liquid biopsy technologies as noninvasive and real-time approached to represent the tumor burden and comprehensively reflect the genetic profile of HCC, e.g. molecular analysis of the tumor-derived fraction of the cell-free DNA (cfDNA) known as circulating tumor DNA (ctDNA), have emerged (Zhang et al. 2021; Roy et al. 2021; Bettegowda et al. 2014). Postoperative ctDNA sequencing has been shown of a great prognostic value in patients with liver cancer (Zhang et al. 2021; Ye et al. 2022). Thus, analysis of ctDNA has many potential applications, including noninvasive tumor genotyping, early detection of tumor recurrence but also detection of minimal residual disease following radiotherapy (Chaudhuri et al. 2015). Adjacent to ctDNA techniques, other biological structures including proteins could lead to personalized therapy based on their identification and alterations in their presence before and after treatment As such, several regulators of the repair responses to liver damage e.g. growth factors and cytokines, including tumor necrosis factor alpha (TNF-α), transforming growth factor beta (TGF-beta) and hedgehog have been associated with fRILI (Lee and Friedman 2011). TGF-beta has been identified to mediate sinusoidal obstruction by deposition of collagen matrix after radiation exposure (Pihusch et al. 2005). Cytokines are highly related to cell-to-cell communication reflecting the local or systemic inflammatory milieu (O’Shea et al. 2002). Since their systemic levels may correlate with organ injury, they can be applied as biomarkers for diseases. Furthermore, they play a key role in tumor escape mechanisms, which are responsible for metastatic outgrowth (Chen et al. 2020; Kany et al. 2019). Although pro-inflammatory cytokines may display anti-cancer activity, they are also responsible for tumor growth. Their local expression levels have been associated with liver integrity, but regarding their systematic levels, cytokine release during therapy or pathology itself can be used for fRILI quantification (Llovet et al. 2021; Conlon et al. 2019; Dranoff 2004). Local therapies are known to induce antigen and cytokine release, as reported in case of inhibitors of the vascular endothelium growth factor (VEGF) and tyrosine kinase that boost immunity and prime tumors for checkpoint inhibition. However, the knowledge about their precise role in fRILI is limited (Llovet et al. 2021; Herranz-Itúrbide et al. 2021). Thus, local therapies can provoke various biological effect caused by the different release pattern of humoral mediators including cytokines and growth factors, which on the one hand enhance the immune response or induce organ damage (e.g. fRILI), and promote tumor progression on the other hand (Llovet et al. 2021; Kim et al. 2013; Fornaro et al. 2014). While VEGF is already in clinical use, several other potential candidates such as nerve growth factor (NGF) have been suggested as markers of tumor progression and potential targets for future innovative therapies (Demir et al. 2016). Thus, identifying further clinical and biological factors that play an important role in the timely diagnose and fRILI progression will improve the understanding of the pathogenesis and potentially maximize the iBT treatment efficacy by reducing the fRILI. Therefore, investigating the correlation between systemic cytokine levels and patient’s outcome, may be useful in understanding their etiology in the clinical course of the pathology. As example, pre- and posttreatment “liquid biopsy” can distinguish between hepatocellular carcinoma patients with progression versus non-progression after radiotherapy (Reiss et al. 2021). With this background, it remains fundamental to understand the role of humoral mediators e.g. cytokines, as potential indicators and/or mediators of local radio sensitivity, radio resistance and distant abscopal effects (Powerski et al. 2020). This latter, radiation-induced effect on the neighboring (bystander) cells as well as fRILI are possibly related to the release of soluble mediators e.g. chemokines and growth factors (Bentzen 2006; Rodemann and Blaese 2007). The aim of this exploratory study was to identify circulating factors in patients undergoing iBT of liver metastases and correlate their levels with both volume and threshold doses of irradiated liver tissue, to potentially propose biomarkers for post-therapeutic fRILI.

## Materials and methods

### Patient cohort

Twenty-two patients (male *n* = 11, female *n* = 11, age 39–80 years) undergoing interstitial HDR brachytherapy of liver metastases (colorectal carcinoma *n* = 16, breast cancer *n* = 4, neuroendocrine tumor *n* = 1, lymphangiosarcoma *n* = 1) were prospectively included in this study. The study was conducted in accordance with the Declaration of Helsinki. Exclusion criteria were clinically ongoing infections and advanced liver cirrhosis with diminished uptake of hepatobiliary contrast agents in MRI. All included patients gave their written informed consent. Prospective data collection and analyses were approved by the local ethics committee. Inclusion criteria were: (i) irresectable liver metastases scheduled for iBT, (ii) no pre-existing liver cirrhosis, (iii) no contraindication to undergo hepatobiliary MRI, (iv) age 18–85 years, (v) ECOG performance status 0 or 1; (vi) no prior irradiation therapy of the same liver lobe.

### Treatment by interstitial brachytherapy

**Fig. 1:**
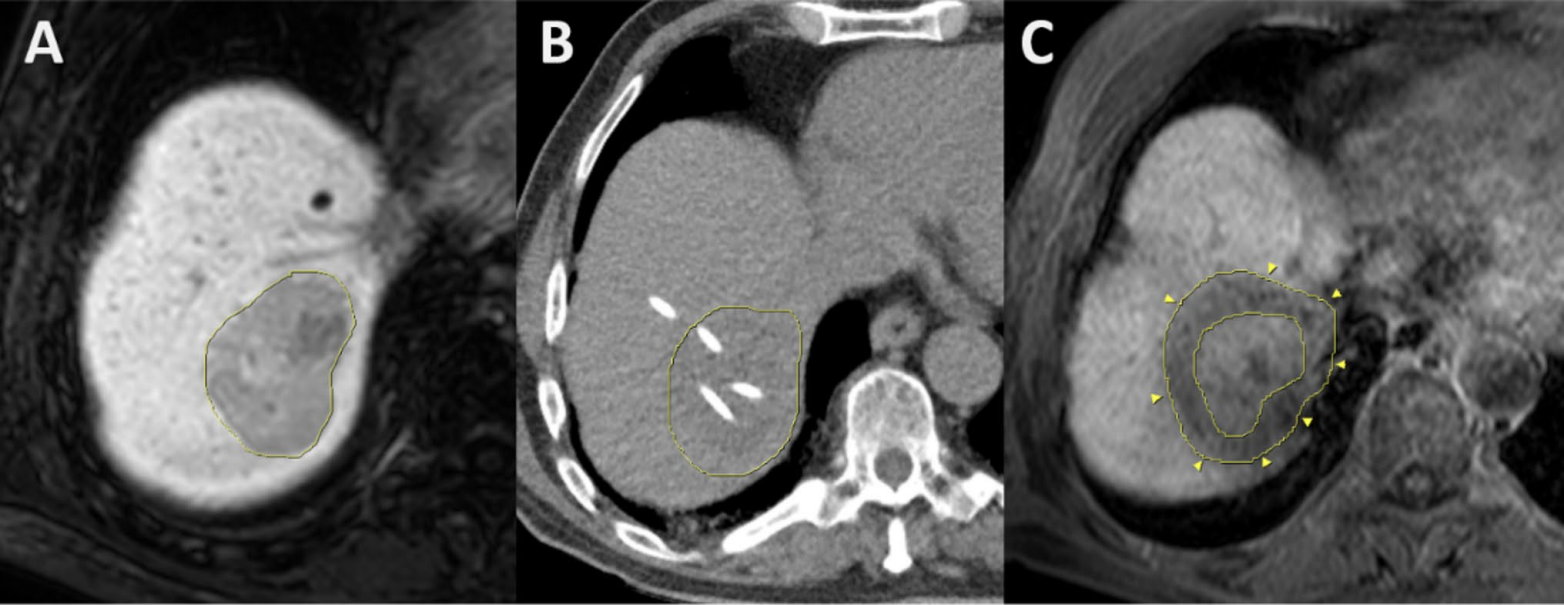
Brachytherapy of a colorectal cancer metastasis. **A** Pre-treatment hepatobiliary magnetic resonance imaging (MRI) depicting a large lesion in liver segment 7. **B** computed tomography-guided insertion of irradiation catheters into the target volume. **C** Follow up hepatobiliary MRI after 6 weeks with shrinkage of the metastasis and additional rim of focal radiation-induced liver injury (arrow heads)

The procedure of interstitial HDR brachytherapy was previously described (Ricke and Wust 2011). Briefly, the 6F catheter sheaths (Terumo Radifocus^®^ Introducer II, Terumo Europe, Leuven, Belgium) and 6F irradiation catheters (afterloading catheter, Primed^®^ medical GmbH, Halberstadt, Germany) were inserted into the liver metastases under computed tomography (CT) fluroscopy utilizing Seldinger’s technique and concomitant conscious sedation with Fentanyl and Midazolam. In complex or larger lesions, multiple catheters may be placed to achieve a sufficient dose distribution and to lower the detrimental radiation effect onto adjacent organs. Then, a single fraction irradiation with an Iridium 192 source was applied in afterloading technique according to a 3D treatment plan (Oncentra^®^ Brachy, Elekta Instrument AB, Stockholm, Sweden). Ablative doses to the planning target volume (PTV) were selected according to the tumor entity (e.g. 25 Gy in colorectal cancer metastases, 15 Gy in metastases of breast cancer or neuroendocrine tumors) (Wieners et al. 2011; Ricke et al. 2010; Schippers et al. 2017). Finally, catheters were removed leaving gelatin sponge in the catheter path to minimize the risk of postinterventional bleeding events. The interstitial brachytherapy data set is described in Fig. 1.

### Data acquisition and follow-up

Regular schedule of interstitial brachytherapy in the Department of Radiology and Nuclear Medicine (Otto-von-Guericke University, Magdeburg, Germany) includes baseline imaging with hepatobiliary MRI, a body CT as well as the laboratory evaluation of liver specific parameters and coagulation status one day prior to irradiation and three days after irradiation. Routine follow-up is assigned every 3 months including imaging and assessment of the standard laboratory parameters. Within this study, hepatobiliary MRI was additionally performed 6 weeks after iBT to detect the maximal extent of fRILI at this time, while the definition of fRILI followed Seidensticker et al. (2014). Blood samples for cytokine analyses were taken after 6 weeks correspondingly. Imaging data of all patients were entirely acquired in a 1.5 T MRI scanner (Achieva, Philips, Best, The Netherlands) including 3D axial THRIVE sequences (T1 high resolution isotropic volume excitation) 20 min after intravenous application of hepatocyte-specific contrast agent Gd-EOB-DTPA (Primovist, Bayer Healthcare, Leverkusen, Germany). MRI data sets where then semi-automatically fused with the CT-based 3D irradiation plan using a point-to-point registration. Afterward, the threshold of fRILI was correlated with the specific isodose. The method was described aby Seidensticker et al. (2014).

### Laboratory analysis of cytokines

Blood samples were obtained one day prior to (baseline) and 6 weeks after irradiation in prechilled citrate tubes (BD Vacutainer, Becton Dickinson Diagnostics, Aalst, Belgium) and were kept on ice. Blood was centrifuged at 2000×*g* for 15 min at 4 °C. Then, plasma was stored at − 80 °C until the sample analyses. Blinded specimens were used for measurements of MCP-1, CX3CL1, VEGF, beta-NGF and TGF-beta by the laboratory of the Experimental Radiology, Department of Radiology and Nuclear Medicine at the University Hospital of the Otto-von-Guericke University Magdeburg using a highly specific commercially available using LEGENDplex Multi-Analyte Flow Assay Kit (BioLegend, San Diego, California) according to manufacturer’s instructions. The measurements and analyses were performed with the BD FACSCanto II™ (BD Heidelberg, Germany). Twenty-two patients were included, and ten healthy volunteers served as controls.

### Statistical analysis

Statistical analyses were performed by IBM SPSS Statistics 22.0^®^. Besides descriptive statistics, changes of cytokine levels in healthy volunteer controls (HV) and tumor patients (TP) before interstitial brachytherapy (pre iBT) and after interstitial brachytherapy (post iBT) were analyzed by the non-parametric Kruskal–Wallis test. Furthermore, spearman correlation analysis was performed to explore correlations between the levels of the assessed humoral mediators and clinical characteristics of the treatment, including tumor and fRILI volumes as well as the isodose. Data are presented as mean ± standard deviation (SD) and in the results given as mean ± standard error of mean (SEM). Statistical significance was assumed for *p* < 0.05.

## Results

### Data description

#### Treatment characteristics

In 22 patients, a total of 30 lesions (1–3 per patient) were ablated by interstitial brachytherapy. Mean liver volume was 1416 ± 382 ml and mean clinical target volume (equalling tumor volume) was 28 ± 35 ml. A D100 of 21.62 ± 4.24 Gy and D90 of 30.46 ± 6.57 Gy was achieved. All patients underwent initial follow-up after 6 weeks including hepatobiliary MRI as well as laboratory evaluation of liver specific and inflammatory parameters. In 19 out of 22 patients (86%), imaging revealed a fRILI with a mean volume of 92 ± 97 ml. Image fusion with the 3D irradiation plan correlated fRILI to a mean hepatic tolerance dose of 14.2 ± 5.7 Gy. A summary of patient and treatment characteristics is given in Table 1.

**Table 1:** Patient and treatment characteristics (*n* = 22)

Variables	*n* (%) or mean ± standard deviation
Age	62.0 ± 10.9 years
Sex (m/w)	*n* = 11 (50%)/*n* = 11 (50%)
Tumor entities	
Colorectal cancer	*n* = 16 (72%)
Breast cancer	*n* = 4 (18%)
Neuroendocrine tumor	*n* = 1 (5%)
Lymphangiosarcoma	*n* = 1 (5%)
Number of lesions	*n* = 30
Liver volume	1416 ± 382 mL
Clinical target volume (CTV)	28 ± 35 mL
Dose to CTV	
D100	21.6 ± 4.2 Gy
D90	30.5 ± 6.6 Gy
Focal radiation-induced liver injury (fRILI)	*n* = 19 (86%)
Threshold isodose	14.2 ± 5.7 Gy
Volume	92 ± 97 mL

#### Humoral mediators in tumor patients pre and post iBT versus healthy volunteer controls

**Fig. 2:**
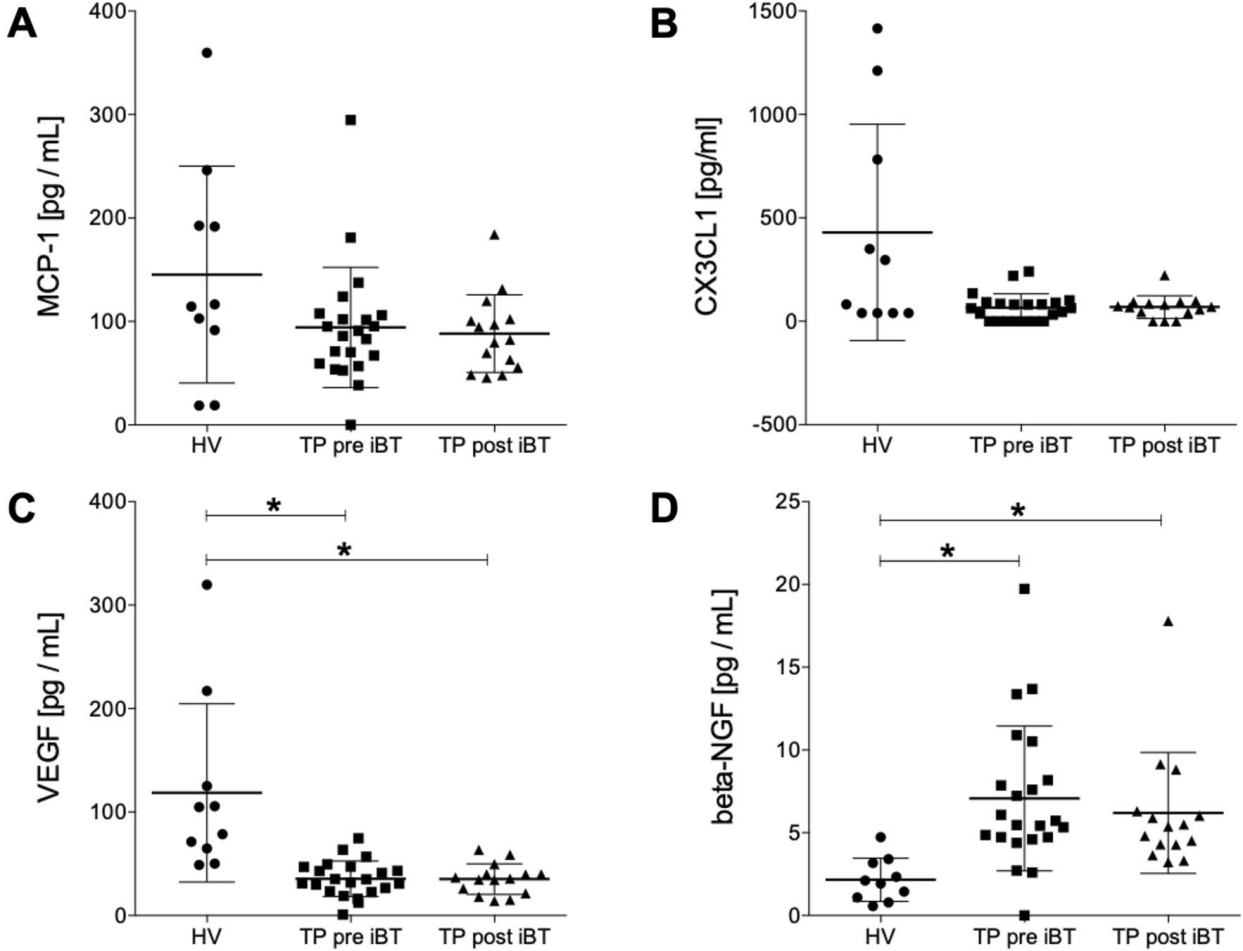
Humoral mediators in tumor patients (TP) pre and post interstitial high dose rate brachytherapy (iBT) versus healthy volunteer controls (HV). Systemic levels of humoral mediators **A** monocyte chemoattractant protein-1 (MCP-1), **B** chemokine (C-X3-C motif) ligand 1 (CX3CL1), **C** vascular endothelial growth factor and **D** beta-nerve growth factor (beta-NGF) are shown. Plasma levels of all humoral mediators were compared from pre iBT (baseline) and post iBT (after 6 weeks) in TP and HV (*n* = 10). Data are presented as mean ± standard error of the mean. *p < 0.05 between the indicated group

The data have shown a trend to decreased MCP-1 levels in TP both pre and post treatment compared to HV; however, no statistical changes were found between the groups (HV: 145.30 ± 33.17 vs. TP pre iBT: 94.30 ± 12.40 vs. TP post iBT: 88.21 ± 9.68 pg/mL; Fig. 2A). Similarly, the CX3CL1 plasma levels demonstrated no significant changes between the three groups (HV: 429.70 ± 165.30 vs. TP pre iBT: 65.84 ± 14.36 vs. TP post iBT: 68.78 ± 14.16 pg/mL; Fig. 2B). Interestingly, the VEGF levels have shown a significant decrease in TP pre and post iBT compared to HV (*p* < 0.05, HV: 118.60 ± 27.26 vs. TP pre iBT: 35.52 ± 3.68 vs. TP post iBT: 35.19 ± 3.81 pg/mL; Fig. 2C). The beta-NGF levels were significantly increased in both TP pre and post iBT compared to HV (*p* < 0.05, HV: 2.16 ± 0.41 vs. TP pre iBT: 7.08 ± 0.93 vs. TP post iBT: 6.20 ± 0.94 pg/mL; Fig. 2D).

#### Correlation analyses of humoral mediators in tumor patients pre and post iBT with the tumor volume

**Fig. 3:**
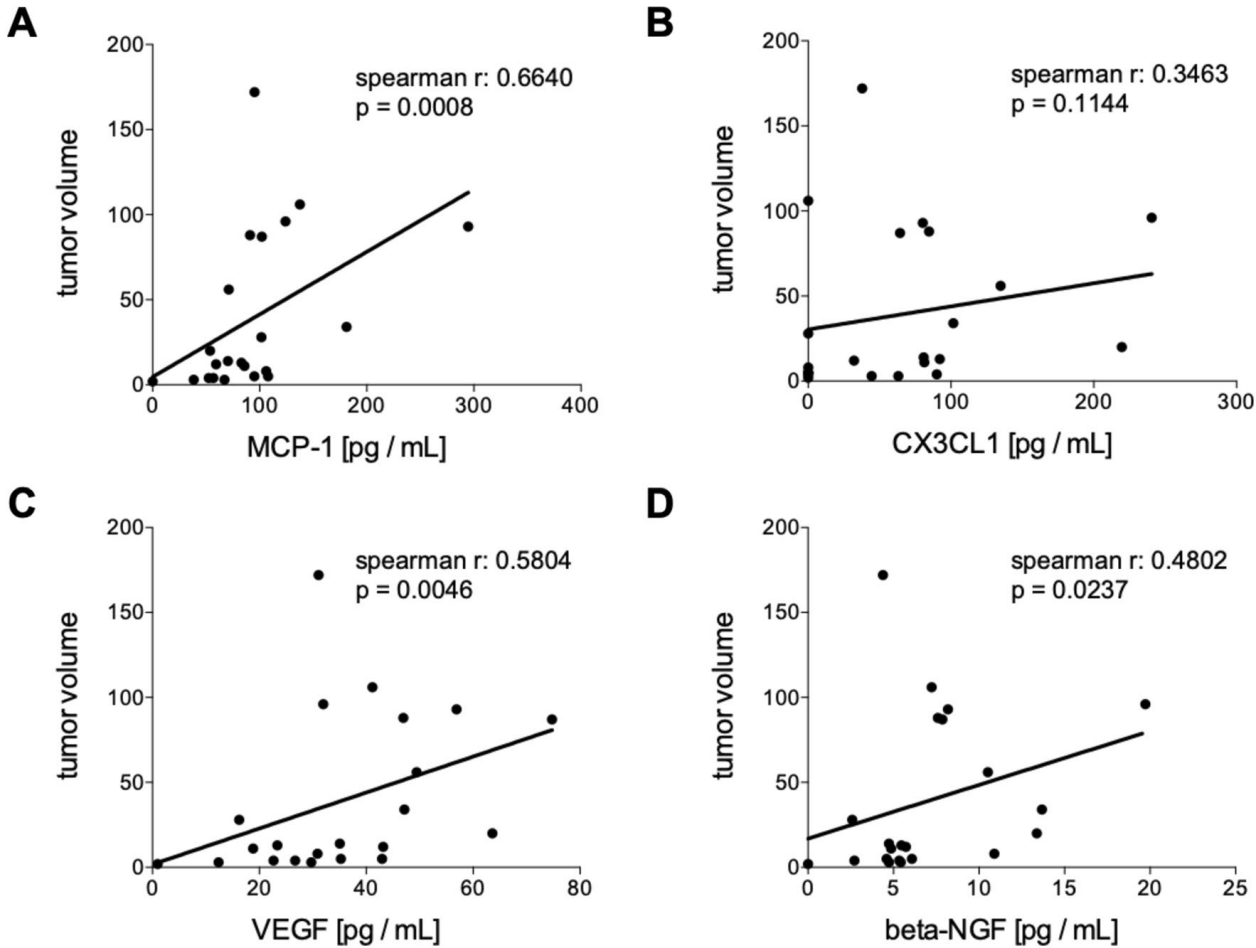
Correlation analyses of humoral mediators in tumor patients (TP) before interstitial high dose rate brachytherapy (pre iBT) with the tumor volume. Spearman correlation analyses between the tumor volume with the systemic levels of **A** monocyte chemoattractant protein-1 (MCP-1), **B** chemokine (C-X3-C motif) ligand 1 (CX3CL1), **C** vascular endothelial growth factor or **D** beta-nerve growth factor (beta-NGF) pre iBT are shown

**Fig. 4:**
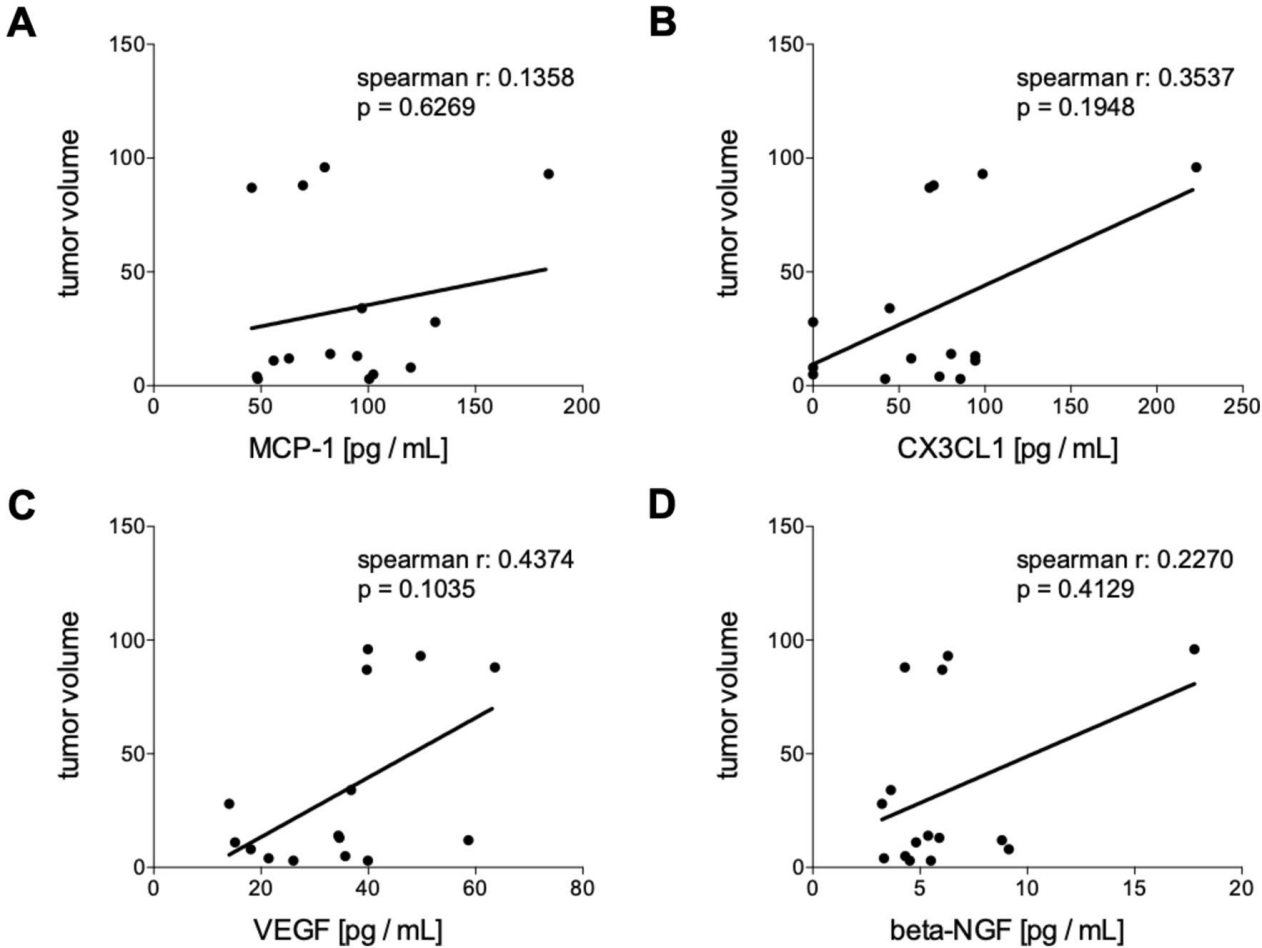
Correlation analyses of humoral mediators in tumor patients (TP) 6 weeks after the interstitial high dose rate brachytherapy (iBT) with the tumor volume. Spearman correlation analyses between the tumor volume with the systemic levels of **A** monocyte chemoattractant protein-1 (MCP-1), **B** chemokine (C-X3-C motif) ligand 1 (CX3CL1), **C** vascular endothelial growth factor or (D) beta-nerve growth factor (beta-NGF) post iBT are shown

Baseline circulating levels of MCP-1, VEGF and beta-NGF have shown significant positive correlations with the hepatic tumor volume (Fig. 3A MCP-1: *r* = 0.6640, *p* = 0.0008. Figure 3C VEGF: *r* = 0.5804, *p* = 0.0046, and Fig. 3D, beta-NGF: *r* = 0.4802, *p* = 0.0237, respectively). CX3CL1 did not correlate with the tumor volume (Fig. 3B). There were no significant correlations between MCP-1, VEGF, beta-NGF and CX3CL1 with the hepatic tumor volume index 6 weeks after interstitial brachytherapy (Fig. 4).

#### Correlation analyses of humoral mediators in tumor patients pre and post iBT with fRILI

**Fig. 5:**
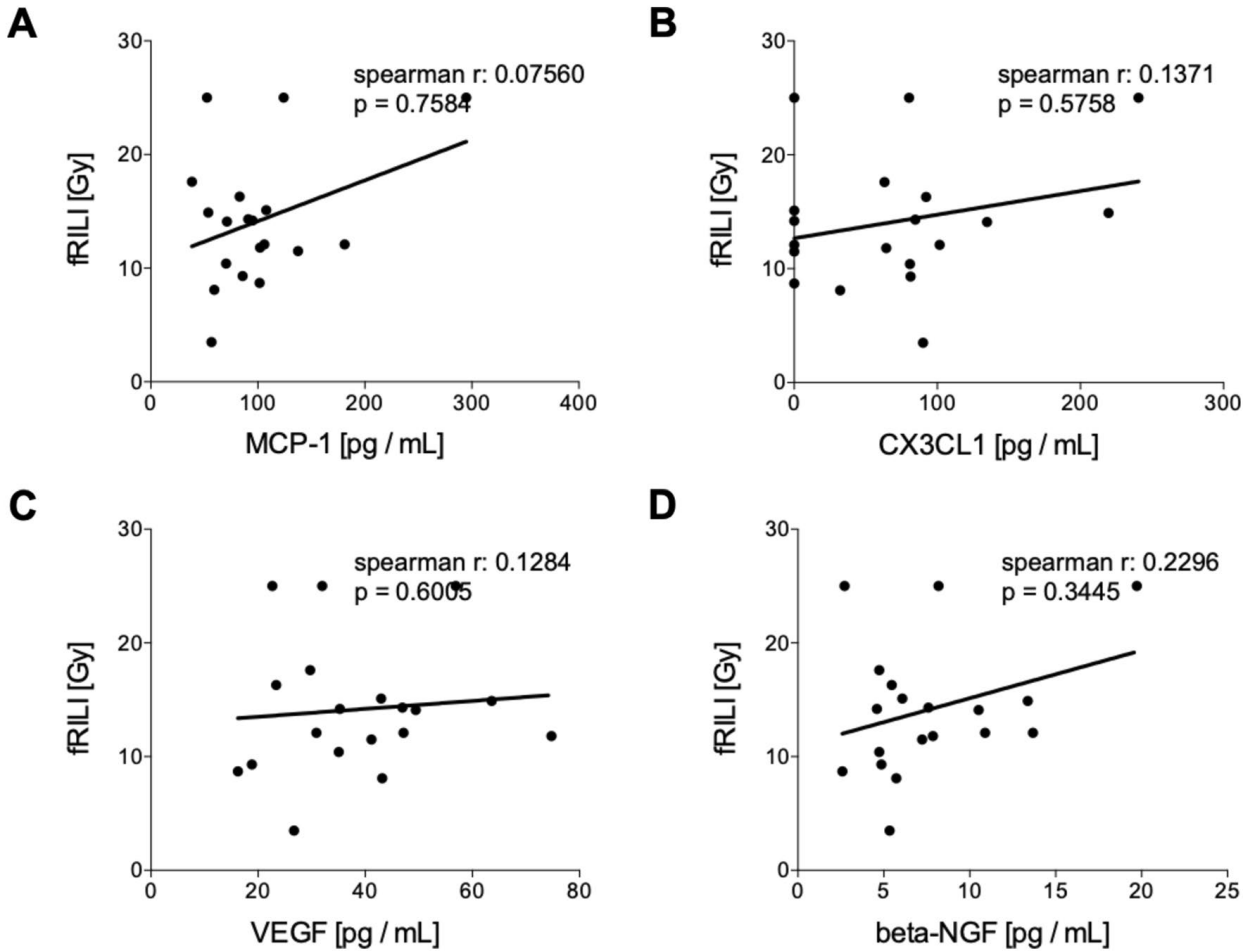
Correlation analyses of humoral mediators in tumor patients (TP) before interstitial high dose rate brachytherapy (pre iBT) with focal radiation-induced liver injury (fRILI). Spearman correlation analyses between fRILI with the systemic levels of **A** monocyte chemoattractant protein-1 (MCP-1), **B** chemokine (C-X3-C motif) ligand 1 (CX3CL1), **C** vascular endothelial growth factor or (D) beta-nerve growth factor (beta-NGF) pre iBT are shown

**Fig. 6:**
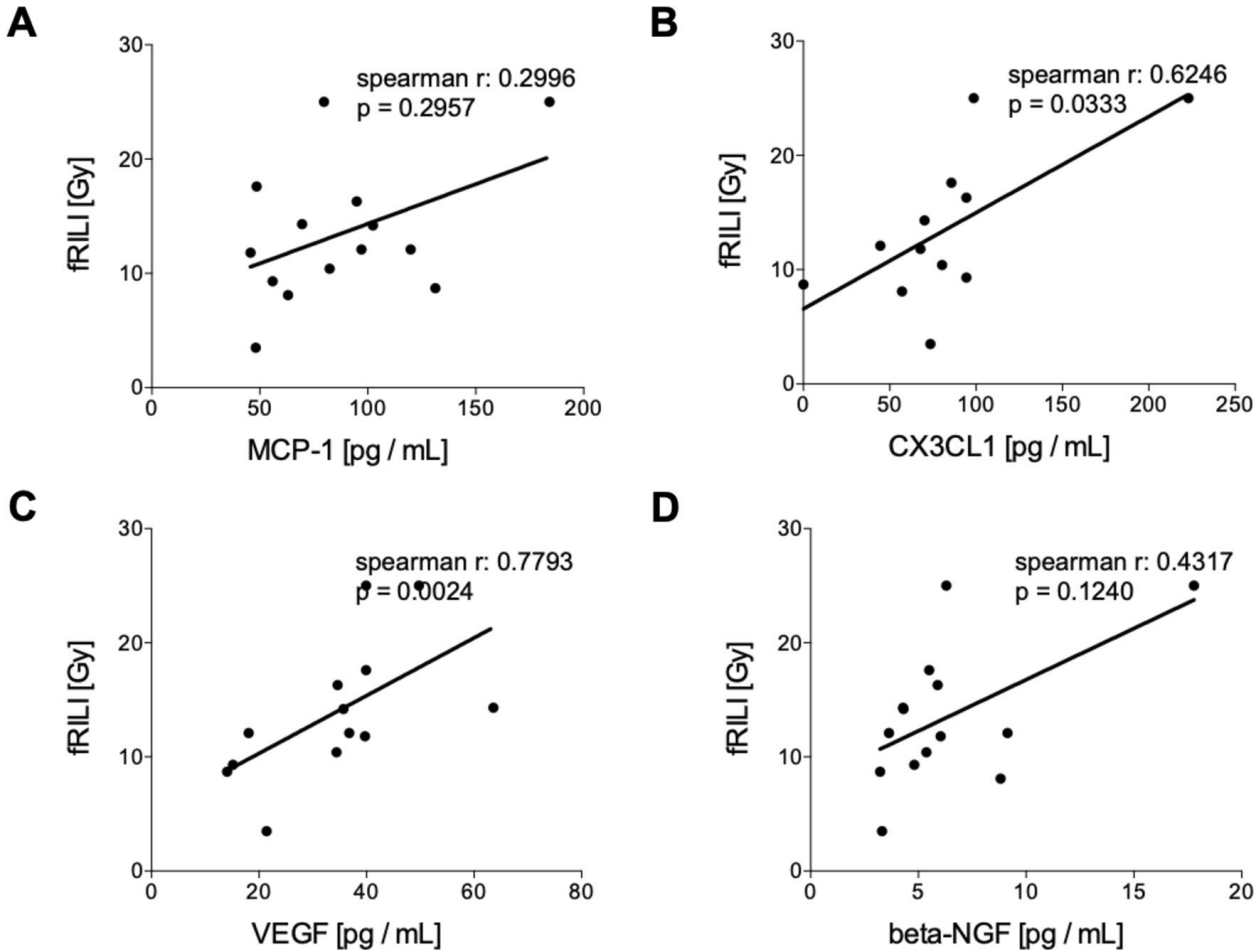
Correlation analyses of humoral mediators in tumor patients (TP) 6 weeks after the interstitial high dose rate brachytherapy (iBT) with focal radiation-induced liver injury (fRILI). Spearman correlation analyses between fRILI with the systemic levels of **A** monocyte chemoattractant protein-1 (MCP-1), **B** chemokine (C-X3-C motif) ligand 1 (CX3CL1), **C** vascular endothelial growth factor or **D** beta-nerve growth factor (beta-NGF) post iBT are shown

Circulating levels of humoral mediators before the treatment did not correlate with fRILI after irradiation (Fig. 5). CX3CL1 and VEGF have shown significant positive correlations with fRILI after treatment (Fig. 5B, CX3CL1: *r* = 0.6246, *p* = 0.0333 and Fig. 5C, VEGF: *r* = 0.7793, *p* = 0.0024, respectively). MCP-1 and beta-NGF levels assessed after interstitial brachytherapy did not correlate with fRILI doses (Fig. 6).

## Discussion

SABR and iBT are irradiation techniques for the therapy of primary and secondary liver malignancies that deliver high-conformal irradiation to target volumes while preserving surrounding healthy liver tissue. However, RILD is still a limiting factor for the therapy application in large tumors and/or multifocal disease and might lead to a dose reduction with subsequent risk of non-ablative treatment. Although the histopathology of RILD is well described and related to VOD or SOS, little is known about the underlying molecular mechanisms, which on the one hand might allow to identifying patients at risk, or on the other hand, may uncover potential targets for preventive medication (Lawrence et al. 1995; Fajardo and Colby 1980). Several findings identified TGF-beta to play a major role in sinusoidal obstruction by promoting proliferation of myofibroblasts, and thereby the deposition of extracellular matrix in hepatic central veins and sinusoids during RILD (Pihusch et al. 2005).

Although hepatobiliary MRI after iBT enables a precise determination of the irradiation damage, we did not find any relationship between circulating TGF-beta levels and either the volume or the threshold isodose of fRILI as the TGF-beta levels were below the detection limit. Thus, our results might suggest that the long-time storage of plasma samples may cause a TGF-beta degradation. Moreover, this could affect the levels of cytokines, since activated platelet fraction, which are the major enhancer of TGF-beta, is reduced using the citrate tube (Assoian and Sporn 1986). This fact should be critically considered since we cannot avoid the inaccurate reflection of circulating levels of TGF-beta ex vivo (Kropf et al. 1997). Interestingly, the lack of TGF-beta in plasma samples might be related to radiation effects directly and locally affecting the liver tissue also. Consequently, the local ablative therapy may have impacted also the local TGF-beta levels, where it exerts its autocrine and paracrine effects (Braun et al. 1988). Importantly, TGF-beta is involved also in liver repair processes (Yang et al. 2016). It has been shown in preclinical studies that the active form of TGF-beta appears to be rapidly sequestered and degraded by the liver (Wakefield et al. 1990). However, we were unable to discern between its active and latent form, and thus, further investigations are required.

In contrast to TGF-beta, significant correlations were found between both MCP-1 and VEGF with the hepatic tumor volume. Furthermore, the data have shown that plasma levels of CXCL1 and VEGF positively correlated with the threshold isodose fRILI. It is known that the tumor growth, metastasis, and veno-occlusive disease (SOD), may depend on the degree of tissue vascularization mediated by VEGF (Kong et al. 2021; Iguchi et al. 2001). However, there is no much information regarding the response to iBT. In our study, we observed that those patients had decreased circulating levels of CX3CL1, VEGF and MCP-1 as compared to healthy donors. Moreover, the correlation between those factors and the tumor volume suggests that the lower baseline circulating levels may indicate the lower tumor volume. A reasonable explanation may be provided by the fact that during the iBT induced tumor cyto-reduction, the bioavailability of VEGF may also have been reduced. This is underlined by the ability of free VEGF to bind to vacant sites, either on its receptors (VEGFR) or binding proteins, which may arise mostly from the tissue damaged by the therapy (Tsai et al. 2021). Taken together, baseline levels of VEGF correlated with the tumor volume, while the post therapy VEGF levels correlated with the fRILI, indicating at a potentially important role of VEGF as a biomarker for therapy outcome.

The current literature indicates that either healthy hepatic or malignant cells are sources of MCP-1 (Wyler et al. 2016; Czaja et al. 1994). Therefore, the positive correlation between MCP-1 and tumor volume reduction may be associated with the iBT, which might consequentially also reduce the circulating protein levels. Similar scenario may also be supported by other chemotactic cytokines such as CX3CL1 (Bazan et al. 1997). Interestingly, CX3CL1 and its receptor can be upregulated in injured human liver (Efsen et al. 2002). Moreover, in hepatocellular carcinoma patients, a good prognosis and low recurrence rates are related to high expression levels of CX3CL1/CX3R1 (Matsubara et al. 2007).

The neuronal tracking can be mediated by various molecules including chemokines and NGF secreted by the tumor microenvironment. Conversely, the latter was found to be associated with wound healing, combined with radiation in vitro (Shi et al. 2003). Therefore, the iBT may provoke specific effects of NGF induction with the aim to protect from radiation and to induce local tissues regeneration. Taken together, further investigations are needed to understand the association between humoral mediators and iBT/fRILI.

### Limitations

Our study has several limitations. One major critical point is the time frame between the clinical therapy application and the laboratory measurements. The stability of cytokines may be affected by long-term storage (de Jager et al. 2009a, b). Therefore, the biomaterial has been processed as soon as possible, and stored at − 80 °C avoiding freezing and thawing processes. This issue needs to be considered in the overall assessment. Moreover, the inclusion criteria did not account whether the tumor patients had natural tolerance or sensitivity to radiation. Therefore, correlating the therapy outcome with biomarkers of liver radiation sensitivity would be advantageous in future. Another limitation is the analysis of the patient cohort with metastases deriving from different primary tumors (e.g. colorectal carcinoma and breast cancer) which might pose a bias as different tumor entities may cause specific microenvironments in involved tissues. This limits the conclusions about tumor volume and circulating cytokines and should be addressed in future studies. Also more frequent time course for the assessment of biomarkers after therapy should be included in a future study. Same applies for the small number of patients included in this study which prohibits detailed statistical tests and/or subgroup analyses. Furthermore, detection of fRILI in hepatobiliary MRI and correlation of with a threshold isodose by 3D image fusion was only reported in a few studies and relies on the diminished uptake of the hepatocyte-specific contrast agent after affecting a single transporting polypeptide (Organic Anion Transporting Polypeptide 1, OATP1). Thus, not all aspects of liver cell functions are reflected by this imaging technique.

## Conclusions

This exploratory study describes several humoral mediators to correlate with the focal hepatic dysfunction after interstitial brachytherapy of liver metastases. Positive correlations of tumor volume and threshold dose of irradiation damage were found for MCP-1 and VEGF as well as NGF and CX3CL, respectively. Therefore, the results from this study suggest that the investigation of humoral mediators in blood samples as “liquid biopsy” from patients may be an appropriate tool to predict side effects such as fRILI after interstitial HDR brachytherapy of liver metastases. However, as a perspective, the patient cohort should be expanded and adapted to homogeneous criteria. We likewise require examining tissue materials from tumor biopsies to characterize the status of the pathology.

## Data Availability

The data can be obtained upon a reasonable request from the corresponding author.

## References

[CR1] Assoian RK, Sporn MB (1986) Type beta transforming growth factor in human platelets: release during platelet degranulation and action on vascular smooth muscle cells. J Cell Biol 102(4):1217–1223 3457014 10.1083/jcb.102.4.1217 PMC2114151

[CR2] Baran M, Yay A, Onder GO, Canturk Tan F, Yalcin B, Balcioglu E (2022) Hepatotoxicity and renal toxicity induced by radiation and the protective effect of quercetin in male albino rats. Int J Radiat Biol. 10.1080/09553002.2022.2033339 35171756 10.1080/09553002.2022.2033339

[CR3] Bayraktar UD, Seren S, Bayraktar Y (2007) Hepatic venous outflow obstruction: three similar syndromes. World J Gastroenterol 13(13):1912–1927 17461490 10.3748/wjg.v13.i13.1912 PMC4146966

[CR4] Bazan JF, Bacon KB, Hardiman G, Wang W, Soo K, Rossi D (1997) A new class of membrane-bound chemokine with a CX3C motif. Nature 385(6617):640–644 9024663 10.1038/385640a0

[CR5] Bentzen SM (2006) Preventing or reducing late side effects of radiation therapy: radiobiology meets molecular pathology. Nat Rev Cancer 6(9):702–713 16929324 10.1038/nrc1950

[CR6] Bettegowda C, Sausen M, Leary RJ, Kinde I, Wang Y, Agrawal N (2014) Detection of circulating tumor DNA in early- and late-stage human malignancies. Sci Transl Med 6(224):224ra24 24553385 10.1126/scitranslmed.3007094 PMC4017867

[CR7] Braun L, Mead JE, Panzica M, Mikumo R, Bell GI, Fausto N (1988) Transforming growth factor beta mRNA increases during liver regeneration: a possible paracrine mechanism of growth regulation. Proc Natl Acad Sci USA 85(5):1539–1543 3422749 10.1073/pnas.85.5.1539 PMC279808

[CR8] Chaudhuri AA, Binkley MS, Osmundson EC, Alizadeh AA, Diehn M (2015) Predicting radiotherapy responses and treatment outcomes through analysis of circulating tumor DNA. Semin Radiat Oncol 25(4):305–312 26384278 10.1016/j.semradonc.2015.05.001 PMC4575502

[CR9] Chen Y, Di C, Zhang X, Wang J, Wang F, Yan JF (2020) Transforming growth factor β signaling pathway: a promising therapeutic target for cancer. J Cell Physiol 235(3):1903–1914 31332789 10.1002/jcp.29108

[CR10] Cheng JCH, Wu JK, Lee PCT, Liu HS, Jian JJM, Lin YM (2004) Biologic susceptibility of hepatocellular carcinoma patients treated with radiotherapy to radiation-induced liver disease. Int J Radiat Oncol Biol Phys 60(5):1502–1509 15590181 10.1016/j.ijrobp.2004.05.048

[CR11] Conlon KC, Miljkovic MD, Waldmann TA (2019) Cytokines in the treatment of cancer. J Interferon Cytokine Res off J Int Soc Interferon Cytokine Res 39(1):6–21 10.1089/jir.2018.0019 PMC6350412 29889594

[CR12] Czaja MJ, Geerts A, Xu J, Schmiedeberg P, Ju Y (1994) Monocyte chemoattractant protein 1 (MCP-1) expression occurs in toxic rat liver injury and human liver disease. J Leukoc Biol 55(1):120–126 8283136 10.1002/jlb.55.1.120

[CR13] de Jager W, Bourcier K, Rijkers GT, Prakken BJ, Seyfert-Margolis V (2009a) Prerequisites for cytokine measurements in clinical trials with multiplex immunoassays. BMC Immunol 10:52 19785746 10.1186/1471-2172-10-52 PMC2761376

[CR14] de Jager W, Prakken B, Rijkers GT (2009b) Cytokine multiplex immunoassay: methodology and (clinical) applications. Methods Mol Biol Clifton NJ 514:119–133 10.1007/978-1-60327-527-9_9 19048217

[CR15] De la Garza-Ramos C, Toskich BB (2021) Radioembolization for the treatment of hepatocellular carcinoma: the road to personalized dosimetry and ablative practice. Semin Interv Radiol 38(4):466–471 10.1055/s-0041-1735571 PMC8497084 34629715

[CR16] Demir IE, Tieftrunk E, Schorn S, Friess H, Ceyhan GO (2016) Nerve growth factor & TrkA as novel therapeutic targets in cancer. Biochim Biophys Acta 1866(1):37–50 27264679 10.1016/j.bbcan.2016.05.003

[CR17] Doi H, Masai N, Uemoto K, Suzuki O, Shiomi H, Tatsumi D (2017) Validation of the liver mean dose in terms of the biological effective dose for the prevention of radiation-induced liver damage. Rep Pract Oncol Radiother J Gt Cancer Cent Poznan Pol Soc Radiat Oncol 22(4):303–309 10.1016/j.rpor.2017.02.011 PMC5422009 28507460

[CR18] Dranoff G (2004) Cytokines in cancer pathogenesis and cancer therapy. Nat Rev Cancer 4(1):11–22 14708024 10.1038/nrc1252

[CR19] Efsen E, Grappone C, DeFranco RMS, Milani S, Romanelli RG, Bonacchi A (2002) Up-regulated expression of fractalkine and its receptor CX3CR1 during liver injury in humans. J Hepatol Juli 37(1):39–47 10.1016/s0168-8278(02)00065-x 12076860

[CR20] Fajardo LF, Colby TV (1980) Pathogenesis of veno-occlusive liver disease after radiation. Arch Pathol Lab Med 104(11):584–588 6893535

[CR21] Fan CQ, Crawford JM (2014) Sinusoidal obstruction syndrome (hepatic veno-occlusive disease). J Clin Exp Hepatol 4(4):332–346 25755580 10.1016/j.jceh.2014.10.002 PMC4298625

[CR22] Fornaro L, Vivaldi C, Caparello C, Sacco R, Rotella V, Musettini G (2014) Dissecting signaling pathways in hepatocellular carcinoma: new perspectives in medical therapy. Future Oncol Lond Engl 10(2):285–304 10.2217/fon.13.181 24490614

[CR23] Hass P, Mohnike K, Kropf S, Brunner TB, Walke M, Albers D (2019) Comparative analysis between interstitial brachytherapy and stereotactic body irradiation for local ablation in liver malignancies. Brachytherapy 18(6):823–828 31522972 10.1016/j.brachy.2019.08.003

[CR24] Herranz-Itúrbide M, Peñuelas-Haro I, Espinosa-Sotelo R, Bertran E, Fabregat I (2021) The TGF-β/NADPH oxidases axis in the regulation of liver cell biology in health and disease. Cells 10(9):2312 34571961 10.3390/cells10092312 PMC8470857

[CR25] Iguchi A, Kobayashi R, Yoshida M, Kobayashi K, Matsuo K, Kitajima I (2001) Vascular endothelial growth factor (VEGF) is one of the cytokines causative and predictive of hepatic veno-occlusive disease (VOD) in stem cell transplantation. Bone Marrow Transplant 27(11):1173–1180 11551028 10.1038/sj.bmt.1703061

[CR26] Kany S, Vollrath JT, Relja B (2019) Cytokines in inflammatory disease. Int J Mol Sci 20(23):E6008 31795299 10.3390/ijms20236008 PMC6929211

[CR27] Kim J, Jung Y (2017) Radiation-induced liver disease: current understanding and future perspectives. Exp Mol Med 49(7):e359 28729640 10.1038/emm.2017.85 PMC5565955

[CR28] Kim MJ, Jang JW, Oh BS, Kwon JH, Chung KW, Jung HS (2013) Change in inflammatory cytokine profiles after transarterial chemotherapy in patients with hepatocellular carcinoma. Cytokine 64(2):516–522 24035756 10.1016/j.cyto.2013.07.021

[CR29] Kong D, Zhou H, Neelakantan D, Hughes CJ, Hsu JY, Srinivasan RR (2021) VEGF-C mediates tumor growth and metastasis through promoting EMT-epithelial breast cancer cell crosstalk. Oncogene 40(5):964–979 33299122 10.1038/s41388-020-01539-x PMC7867573

[CR30] Kropf J, Schurek JO, Wollner A, Gressner AM (1997) Immunological measurement of transforming growth factor-beta 1 (TGF-beta1) in blood; assay development and comparison. Clin Chem 43(10):1965–1974 9342020

[CR31] Lawrence TS, Robertson JM, Anscher MS, Jirtle RL, Ensminger WD, Fajardo LF (1995) Hepatic toxicity resulting from cancer treatment. Int J Radiat Oncol Biol Phys 31(5):1237–1248 7713785 10.1016/0360-3016(94)00418-K

[CR32] Lee UE, Friedman SL (2011) Mechanisms of hepatic fibrogenesis. Best Pract Res Clin Gastroenterol 25(2):195–206 21497738 10.1016/j.bpg.2011.02.005 PMC3079877

[CR33] Liang SX, Huang XB, Zhu XD, Zhang WD, Cai L, Huang HZ (2011) Dosimetric predictor identification for radiation-induced liver disease after hypofractionated conformal radiotherapy for primary liver carcinoma patients with Child-Pugh Grade A cirrhosis. Radiother Oncol J Eur Soc Ther Radiol Oncol 98(2):265–269 10.1016/j.radonc.2010.10.014 21056489

[CR34] Llovet JM, De Baere T, Kulik L, Haber PK, Greten TF, Meyer T (2021) Locoregional therapies in the era of molecular and immune treatments for hepatocellular carcinoma. Nat Rev Gastroenterol Hepatol 18(5):293–313 33510460 10.1038/s41575-020-00395-0

[CR35] Manchec B, Kokabi N, Narayanan G, Niekamp A, Peña C, Powell A (2021) Radioembolization of secondary hepatic malignancies. Semin Interv Radiol 38(4):445–452 10.1055/s-0041-1732318 PMC8497082 34629712

[CR36] Matsubara T, Ono T, Yamanoi A, Tachibana M, Nagasue N (2007) Fractalkine-CX3CR1 axis regulates tumor cell cycle and deteriorates prognosis after radical resection for hepatocellular carcinoma. J Surg Oncol 95(3):241–249 17323338 10.1002/jso.20642

[CR37] O’Shea JJ, Ma A, Lipsky P (2002) Cytokines and autoimmunity. Nat Rev Immunol 2(1):37–45 11905836 10.1038/nri702

[CR38] Pan CC, Kavanagh BD, Dawson LA, Li XA, Das SK, Miften M (2010) Radiation-associated liver injury. Int J Radiat Oncol Biol Phys 76(3 Suppl):S94-100 20171524 10.1016/j.ijrobp.2009.06.092 PMC4388033

[CR39] Pihusch V, Pihusch M, Penovici M, Kolb HJ, Hiller E, Pihusch R (2005) Transforming growth factor beta-1 released from platelets contributes to hypercoagulability in veno-occlusive disease following hematopoetic stem cell transplantation. Thromb Res 116(3):233–240 15935832 10.1016/j.thromres.2004.12.010

[CR40] Powerski M, Drewes R, Omari J, Relja B, Surov A, Pech M (2020) Intra-hepatic abscopal effect following radioembolization of hepatic metastases. Cardiovasc Intervent Radiol 43(11):1641–1649 32808201 10.1007/s00270-020-02612-4 PMC7591411

[CR41] Reiss KA, Wattenberg MM, Damjanov N, Prechtel Dunphy E, Jacobs-Small M, Lubas MJ (2021) A pilot study of galunisertib plus stereotactic body radiotherapy in patients with advanced hepatocellular carcinoma. Mol Cancer Ther 20(2):389–397 33268571 10.1158/1535-7163.MCT-20-0632 PMC7867621

[CR42] Ricke J, Wust P (2011) Computed tomography-guided brachytherapy for liver cancer. Semin Radiat Oncol 21(4):287–293 21939858 10.1016/j.semradonc.2011.05.005

[CR43] Ricke J, Mohnike K, Pech M, Seidensticker M, Rühl R, Wieners G (2010) Local response and impact on survival after local ablation of liver metastases from colorectal carcinoma by computed tomography-guided high-dose-rate brachytherapy. Int J Radiat Oncol Biol Phys 78(2):479–485 20304566 10.1016/j.ijrobp.2009.09.026

[CR44] Robbins MEC, Zhao W (2004) Chronic oxidative stress and radiation-induced late normal tissue injury: a review. Int J Radiat Biol 80(4):251–259 15204702 10.1080/09553000410001692726

[CR45] Rodemann HP, Blaese MA (2007) Responses of normal cells to ionizing radiation. Semin Radiat Oncol 17(2):81–88 17395038 10.1016/j.semradonc.2006.11.005

[CR46] Romesser PB, Neal BP, Crane CH (2021) External beam radiation therapy for liver metastases. Surg Oncol Clin N Am 30(1):159–173 33220803 10.1016/j.soc.2020.08.006

[CR47] Roy D, Lucci A, Ignatiadis M, Jeffrey SS (2021) Cell-free circulating tumor DNA profiling in cancer management. Trends Mol Med 27(10):1014–1015 34312074 10.1016/j.molmed.2021.07.001

[CR48] Sangro B, Gil-Alzugaray B, Rodriguez J, Sola I, Martinez-Cuesta A, Viudez A (2008) Liver disease induced by radioembolization of liver tumors: description and possible risk factors. Cancer 112(7):1538–1546 18260156 10.1002/cncr.23339

[CR49] Schippers AC, Collettini F, Steffen IG, Wieners G, Denecke T, Pavel M (2017) Initial experience with CT-guided high-dose-rate brachytherapy in the multimodality treatment of neuroendocrine tumor liver metastases. J Vasc Interv Radiol JVIR 28(5):672–682 27645463 10.1016/j.jvir.2016.07.011

[CR50] Seidensticker M, Seidensticker R, Damm R, Mohnike K, Pech M, Sangro B (2014) Prospective randomized trial of enoxaparin, pentoxifylline and ursodeoxycholic acid for prevention of radiation-induced liver toxicity. PLoS ONE 9(11):e112731 25393877 10.1371/journal.pone.0112731 PMC4231047

[CR51] Shi CM, Qu JF, Cheng TM (2003) Effects of the nerve growth factor on the survival and wound healing in mice with combined radiation and wound injury. J Radiat Res (tokyo) 44(3):223–228 14646225 10.1269/jrr.44.223

[CR52] Shulman HM, Hinterberger W (1992) Hepatic veno-occlusive disease–liver toxicity syndrome after bone marrow transplantation. Bone Marrow Transplant 10(3):197–214 1422475

[CR53] Tsai TH, Chen YJ, Wang LY, Hsieh CH (2021) Effect of synchronous versus sequential regimens on the pharmacokinetics and biodistribution of regorafenib with irradiation. Pharmaceutics 13(3):386 33805831 10.3390/pharmaceutics13030386 PMC8035703

[CR54] Van Cutsem E, Cervantes A, Adam R, Sobrero A, Van Krieken JH, Aderka D (2016) ESMO consensus guidelines for the management of patients with metastatic colorectal cancer. Ann Oncol 27(8):1386–1422 27380959 10.1093/annonc/mdw235

[CR55] Vogel A, Martinelli E, Vogel A, Cervantes A, Chau I, Daniele B (2021) Updated treatment recommendations for hepatocellular carcinoma (HCC) from the ESMO clinical practice guidelines. Ann Oncol 32(6):801–805 33716105 10.1016/j.annonc.2021.02.014

[CR56] Wakefield LM, Winokur TS, Hollands RS, Christopherson K, Levinson AD, Sporn MB (1990) Recombinant latent transforming growth factor beta 1 has a longer plasma half-life in rats than active transforming growth factor beta 1, and a different tissue distribution. J Clin Investig 86(6):1976–1984 2254455 10.1172/JCI114932 PMC329834

[CR57] Wieners G, Mohnike K, Peters N, Bischoff J, Kleine-Tebbe A, Seidensticker R (2011) Treatment of hepatic metastases of breast cancer with CT-guided interstitial brachytherapy—a phase II-study. Radiother Oncol J Eur Soc Ther Radiol Oncol 100(2):314–319 10.1016/j.radonc.2011.03.005 21497930

[CR58] Wyler SL, D’Ingillo SL, Lamb CL, Mitchell KA (2016) Monocyte chemoattractant protein-1 is not required for liver regeneration after partial hepatectomy. J Inflamm Lond Engl 13(1):28 10.1186/s12950-016-0136-1 PMC4994209 27555804

[CR59] Yahyapour R, Motevaseli E, Rezaeyan A, Abdollahi H, Farhood B, Cheki M (2018) Reduction-oxidation (redox) system in radiation-induced normal tissue injury: molecular mechanisms and implications in radiation therapeutics. Clin Transl Oncol off Publ Fed Span Oncol Soc Natl Cancer Inst Mex 20(8):975–988 10.1007/s12094-017-1828-6 29318449

[CR60] Yang AT, Hu DD, Wang P, Cong M, Liu TH, Zhang D (2016) TGF-β1 induces the dual regulation of hepatic progenitor cells with both anti- and proliver fibrosis. Stem Cells Int 2016:1492694 26839553 10.1155/2016/1492694 PMC4709730

[CR61] Ye K, Fan Q, Yuan M, Wang D, Xiao L, Long G (2022) Prognostic value of postoperative circulating tumor DNA in patients with early- and intermediate-stage hepatocellular carcinoma. Front Oncol 12:834992 35311090 10.3389/fonc.2022.834992 PMC8931326

[CR62] Zhang Y, Liu Z, Ji K, Li X, Wang C, Ren Z (2021) Clinical application value of circulating cell-free DNA in hepatocellular carcinoma. Front Mol Biosci 8:736330 34660697 10.3389/fmolb.2021.736330 PMC8511426

